# Inhibitory axons are targeted in hippocampal cell culture by anti-Caspr2 autoantibodies associated with limbic encephalitis

**DOI:** 10.3389/fncel.2015.00265

**Published:** 2015-07-09

**Authors:** Delphine Pinatel, Bruno Hivert, José Boucraut, Margaux Saint-Martin, Véronique Rogemond, Lida Zoupi, Domna Karagogeos, Jérôme Honnorat, Catherine Faivre-Sarrailh

**Affiliations:** ^1^Aix Marseille Université, CNRS, Centre de Recherche en Neurobiologie et Neurophysiologie de Marseille, CRN2M-UMR7286, Faculté de Médecine NordMarseille, France; ^2^Laboratoire d’Immunologie et d’Immunopathologie, AP-HM, Hôpital de la ConceptionMarseille, France; ^3^French Reference Center on Paraneoplastic Neurological Syndrome, Hospices Civils de Lyon, Hôpital NeurologiqueBron, France; ^4^INSERM U1028 – CNRS UMR 5292, Lyon Neuroscience Research CenterLyon, France; ^5^Université de Lyon – Université Claude Bernard Lyon 1Lyon, France; ^6^Institute of Molecular Biology and Biotechnology – Foundation for Research and Technology, University of CreteHeraklion, Greece

**Keywords:** CNTNAP2, TAG-1, contactin 2, voltage-gated potassium channels, autoimmunity, interneurons, cell adhesion

## Abstract

Contactin-associated protein-like 2 (Caspr2), also known as CNTNAP2, is a cell adhesion molecule that clusters voltage-gated potassium channels (Kv1.1/1.2) at the juxtaparanodes of myelinated axons and may regulate axonal excitability. As a component of the Kv1 complex, Caspr2 has been identified as a target in neuromyotonia and Morvan syndrome, but also in some cases of autoimmune limbic encephalitis (LE). How anti-Caspr2 autoimmunity is linked with the central neurological symptoms is still elusive. In the present study, using anti-Caspr2 antibodies from seven patients affected by pure LE, we determined that IgGs in the cerebrospinal fluid of four out seven patients were selectively directed against the N-terminal Discoïdin and LamininG1 modules of Caspr2. Using live immunolabeling of cultured hippocampal neurons, we determined that serum IgGs in all patients strongly targeted inhibitory interneurons. Caspr2 was highly detected on GAD65-positive axons that are surrounding the cell bodies and at the VGAT-positive inhibitory presynaptic contacts. Functional assays indicated that LE autoantibodies may induce alteration of Gephyrin clusters at inhibitory synaptic contacts. Next, we generated a Caspr2-Fc chimera to reveal Caspr2 receptors on hippocampal neurons localized at the somato-dendritic compartment and post-synapse. Caspr2-Fc binding was strongly increased on TAG-1-transfected neurons and conversely, Caspr2-Fc did not bind hippocampal neurons from TAG-1-deficient mice. Our data indicate that Caspr2 may participate as a cell recognition molecule in the dynamics of inhibitory networks. This study provides new insight into the potential pathogenic effect of anti-Caspr2 autoantibodies in central hyperexcitability that may be related with perturbation of inhibitory interneuron activity.

## Introduction

Contactin-associated protein-like 2 Caspr2 (also known as CNTNAP2) is a CAM that belongs to the Neurexin family and is associated with both neuropsychiatric disorders and autoimmune diseases. One function of Caspr2 has been well characterized as a component of the juxtaparanodes of myelinated fibers in the CNS and PNS (Poliak et al., 2003). Caspr2 extracellular domain interacts with contactin 2/TAG-1, a glycosyl-phosphatidyl-inositol anchored Ig-CAM expressed by both axonal and facing glial membranes (Traka et al., 2003). Caspr2 cytoplasmic tail contains a binding site for the cytoskeleton adaptor protein 4.1B and a C-terminal PDZ binding sequence (Horresh et al., 2008). The Caspr2 complex mediates the clustering of VGKCs, mainly Kv1.1 and Kv1.2 at juxtaparanodes (Poliak et al., 2003; Traka et al., 2003). The role attributed to these channels is to stabilize conduction at the nodes of Ranvier, avoid repetitive firing and help to maintain the internodal resting potential (Rasband, 1998; Devaux and Gow, 2008). Knock-out mice for either Caspr2, TAG-1, or protein 4.1B display diffused of Kv1.1/1.2 along the internode, albeit, the mis-localization of Kv1 channels does not affect nerve conduction (Poliak et al., 2003; Traka et al., 2003; Cifuentes-Diaz et al., 2011). Caspr2 also co-localizes with the Kv1.1/Kv1.2 channels at the axon initial segment and may regulate axonal excitability at this site (Inda et al., 2006; Ogawa et al., 2008).

Apart from its well-known function in Kv1 channel clustering at juxtaparanodes, Caspr2 may also act as a cell recognition molecule during development and synaptic network formation. Caspr2-deficient mice show a defect in the migration of cortical neurons and a reduction in the number of GABAergic interneurons which are associated with an epileptic phenotype and autism-related behaviors (Penagarikano et al., 2011). RNAi-mediated knockdown of Caspr2 affects synaptic organization and function in culture (Anderson et al., 2012). Mutations of the Caspr2 gene (*cntnap2*) have been unambiguously associated with neuropsychiatric disorders, such as developmental language impairment and autistic spectrum disorders (Strauss et al., 2006; Bakkaloglu et al., 2008; Penagarikano and Geschwind, 2012; Rodenas-Cuadrado et al., 2013). However, the altered neuronal functions underlying these disorders remain elusive.

Numerous studies have implicated the VGKC-complex as an autoimmune target in generalized neuromyotonia, persistent facial myokymia, Morvan’s syndrome, and in LE (Vincent et al., 2004). Recent studies revealed that in most patients with anti-VGKC-complex antibodies, the immune targets are in fact Leucine-rich glioma inactivated 1 (LGI1), a secreted protein associated with presynaptic Kv1 channels (Lai et al., 2010) or the juxtaparanodal CAMs, Caspr2 and TAG-1 (Irani et al., 2010; Lancaster et al., 2011). In the present study, we used anti-Caspr2 IgGs of patients with pure LE to label cultured hippocampal neurons and characterize targeted cell types and subcellular compartments. In addition, we generated a Caspr2-Fc chimera to analyze the distribution of Caspr2 binding sites on hippocampal neurons. We showed that Caspr2 is mainly expressed by inhibitory axons and may participate to trans-synaptic adhesion complexes. This study provides new insight into the potential pathogenic effect of anti-Caspr2 autoantibodies in central hyperexcitability that may be related with perturbation of inhibitory synaptic transmission.

## Materials and Methods

### Constructs

The pCDNA3-Caspr2-HA construct encodes human Caspr2 with the HA epitope inserted downstream the signal peptide between the residues Trp26 and Thr27 (Bel et al., 2009). Caspr2-mcherry was generated by PCR amplification and insertion into the EcoR1-BamH1 sites of pmCherry-N1 vector. The Caspr2-HA deleted constructs, Caspr2Δ1 (Δ32-361), Caspr2Δ2 (Δ362-600), Caspr2Δ3 (Δ600-950), Caspr2Δ4 (Δ955–1169) were generated by QuickChange mutagenesis (Agilent Technologies). The Caspr2-Discoïdin-LamininG1, Caspr2-Discoïdin, Caspr2-LamininG1 (**Figure 2A**) were obtained using reverse PCR on HA-tagged full length Caspr2 plasmid and the fragment fusion was performed using the In-Fusion kit (Clontech). The Caspr2-Fc construct was generated by PCR amplification of Caspr2 extracellular domain (amino acids 1–1242) and insertion into the Kpn1-Not1 cloning sites of pIg-plus vector. The human TAG-1-GFP construct was generated by inserting GFP downstream the signal peptide. PCR amplified products were verified by sequencing (Beckman Coulter Genomics). Plasmids encoding human LGI1, ADAM22, ADAM23 were purchased from Origene. Gephyrin-GFP is a kind gift of Dr. F. Ango.

### Patient’s Serum and CSF

The presence of anti-Caspr2 autoantibodies was assessed using the patient’s cerebrospinal fluid (CSF) as previously described (Viaccoz et al., 2014). Patients were considered positive when positive staining of a cell-based assay with human embryonic kidney cells (HEK-293) cells overexpressing the Caspr2 protein was observed. After the identification of anti-Caspr2 antibodies by the French PNS Reference Center, serum and CSF samples were frozen and conserved at -80°C. A written consent was obtained from all patients, and the use of samples for this study was approved by the institutional review board of the University Claude Bernard Lyon 1/Hospices Civils de Lyon. The patient’s clinical data were prospectively collected at least twice a year by phone or mail.

### Antibodies and Immunofluorescence Staining

The IgGs from patients’ sera were purified using the Melon gel kit (Pierce). Rabbit anti-Caspr and rat anti-Gliomedin were described previously (Labasque et al., 2011), rabbit anti-MAP2 was a gift from Dr. J. F. Leterrier, rabbit anti-AnkyrinG from Dr. G. Alcaraz, and rabbit anti-vGLUT1 from Dr. S. El Mestikawy. The mouse anti-GAD65 mAb was obtained from Developmental Studies Hybridoma Bank, University of Iowa and anti-Kv1.2 (clone K14/16) mAb from UC Davis/NIH NeuroMab Facility, UCDavis, mouse anti-tau mAb was purchased from Sigma, mouse anti-Synaptophysin mAb from Chemicon, guinea-pig anti-VGAT from Synaptic Systems and rat anti-HA mAb was purchased from Roche. AlexaFluor488-, 568-, and 647-conjugated secondary antibodies were obtained from Molecular Probes. Immunostaining with IgGs from LE patients was performed on live cells diluted 1:500 in culture medium for 30–60 min. Cells were fixed with 4% paraformaldehyde in PBS for 10 min and permeabilized with 0.1% Triton-X100 for 10 min. Immunofluorescence staining was performed using rabbit anti-MAP2 (1:2000), rabbit anti-vGLUT1 (1:100), rabbit anti-AnkyrinG (1:500), or guinea-pig anti-VGAT (1:400) antibodies, or mouse anti-tau (1:500), anti-Kv1.2 (1:500), anti-GAD65 (1:200), or anti-Synaptophysin (1:200) mAbs, and with secondary antibodies diluted in PBS containing 3% bovine serum albumin. After washing in PBS, cells were mounted in Mowiol (Calbiochem).

### Flow Cytometry and Isotyping

HEK-293 cells were transfected with Caspr2-HA, harvested and double-labeled using anti-HA mAb and IgGs from LE patients and secondary antibodies conjugated with FITC or Phycoerythrin (Beckman Coulter). FITC-conjugated anti-human IgG1, IgG2, IgG3, and IgG4 were purchased from Bindingsite. Cells were washed and fixed with 2% paraformaldehyde and analyzed on FACSCanto with the CellQuest software (Becton Dickinson).

### Cell Culture

Cell culture media and reagents were from Invitrogen. Neuroblastoma N2a cells and HEK-293 cells were grown in DMEM containing 10% fetal calf serum and were transiently transfected using jet PEI (Polyplus transfection, Ozyme). Caspr2-Fc, control and deletion mutants were produced in the supernatant of transfected HEK-293 cells and affinity purified using Protein-A Sepharose. Transfected N2a cells and hippocampal neurons were incubated with Caspr2-Fc (10 μg/ml) pre-clustered with Alexa488 or 568 conjugated anti-human Fc (50 μg/ml) for 30 min at 37°C. Primary hippocampal cell cultures were performed from embryonic day 18-Wistar rats. Hippocampi were collected in Hanks’ balanced salt solution, dissociated with trypsin and plated at a density of 1.2.10^5^ cells/cm^2^ on poly L-lysine coated coverslips. The hippocampal neurons were cultured in Neurobasal supplemented with 2% B-27, 1% penicillin-streptomycin, and 0.3% glutamine in a humidified atmosphere containing 5% CO_2_ at 37°C. Hippocampal neurons were transfected using Lipofectamine 2000 with Gephyrin-GFP or GFP at DIV 14, or with TAG-1-GFP, LGI1-GFP, ADAM22, and ADAM23 at DIV8. Hippocampal cell cultures were prepared from embryonic day 16 C57BL6 wild-type and *Tag-1^-/-^* mice (Traka et al., 2003) with the same protocol. For functional perturbing assays, DIV17 neurons transfected with Gephyrin-GFP were incubated for 1 h at 37°c with culture medium, control, LE5 or LE6 IgGs using 1/100 dilution in 100 μl volume before fixation and immunostaining for GAD65. Experiments were performed in duplicate and four coverslips analyzed under each condition. All animal experiments were carried out according to the animal care and experimentation committee rules approved by CNRS.

### Confocal Microscopy and Image Analysis

Image acquisition was performed on a Zeiss laser-scanning microscope equipped with 63 × 1.32 NA oil-immersion objective. Images of GFP or AlexaFluor-stained cells were obtained using the 488 nm band of an Argon laser and the 568 and 647 nm bands of a solid state laser for excitation. Fluorescence images were collected automatically with an average of two-frame scans and collected as frame-by-frame sequential series for tiles. To quantify the number of inhibitory pre-synaptic contacts immunostained for Caspr2, we estimated the number of GAD65 clusters that were positive or negative for Caspr2 along 25 μm-dendrite lengths (*n* = 14 neurons). To quantify the number of post-synaptic contacts labeled for Caspr2-Fc, we estimated the number of Synaptophysin clusters contacting the shaft and spines that were positive or negative for Caspr2-Fc along 50 μm-dendrite lengths (*n* = 21 dendrites, 7 neurons) using the image-J software. To quantify the number of synaptic and total Gephyrin-GFP clusters per neuron, we used Imaris as software (BitplaneAG, Switzerland) with automatic detection of objects in 3-dimensional space using six *z*-stack projections. The *“spot”* tool of surpass function was used to detect the GAD65 pre-synaptic clusters and post-synaptic Gephyrin-GFP clusters and the same segmentation threshold was used for all the images in each channel. The intracellular aggregates of Gephyrin-GFP (spot diameter >0.6 μm) were removed. We selected the post-synaptic spots opposed to pre-synaptic spots within a 0.6 μm distance with the “*co-localize spots*” option. The ratio of synaptic relative to total Gephyrin clusters and the number of synaptic Gephyrin clusters per neuron were determined under each condition. The total number GAD65 clusters contacting the somato-dendritic compartment was determined using the *“find spots close to surface”* tool. To analyze the effect of incubation with control and LE IgGs, data were pooled from two independent cultures (four coverslips, *n* = 23–36 neurons analyzed under each condition) and significant differences were determined using ANOVA followed by Fisher’s test.

## Results

### Autoantibodies to Caspr2 in LE Bind Hippocampal Neurons in Culture

We identified Caspr2 as a target antigen in a series of seven patients with LE. The clinical features in **Table 1** indicate that these patients showed pure LE characterized by confusion, amnesia, and seizures, without neuromyotonia. All the sera (named LE1-LE7) were reactive for dendrotoxin-precipitated VGKC as analyzed using radio-immunoassays, negative for LGI1 and reacted against Caspr2 at high titer as assayed using cell binding assays and flow cytometry (**Table 1**).

**Table 1:** Basic epidemiological, immunological, and clinical features of LE patients with antibodies against Caspr2.

Patient	Age (gender)	Titers	Clinical features	Treatment with improvement
LEI	73 (M)	1/51,200	limbic encephalitis, confusion, seizures, prostate cancer	IVIg
LE2	62 (M)	1/12,800	limbic encephalitis, confusion, temporal seizures, prostate cancer	IVIg
LE3	60 (M)	1/6,400	memory disturbances, thyroid cancer with metastasis	plasmapheresis
LE4	71 (M)	1/51,200	memory disturbances, temporal seizures	corticosteroids, mycophenolate, mofetil
LE5	64 (M)	1/51,200	limbic encephalitis, confusion, ataxia, seizures	corticosteroids
LE6	60 (M)	1/51,200	limbic encephalitis, confusion, amnesia, sub-clinical seizures	corticosteroids
LE7	72 (M)	1/12,800	limbic encephalitis, confusion, amnesia, seizures	corticosteroids, anti-convulsant

*These patients did not present neuromyotonia. The titer of serum IgGs was determined using flow cytometry.*

**FIGURE 1:**
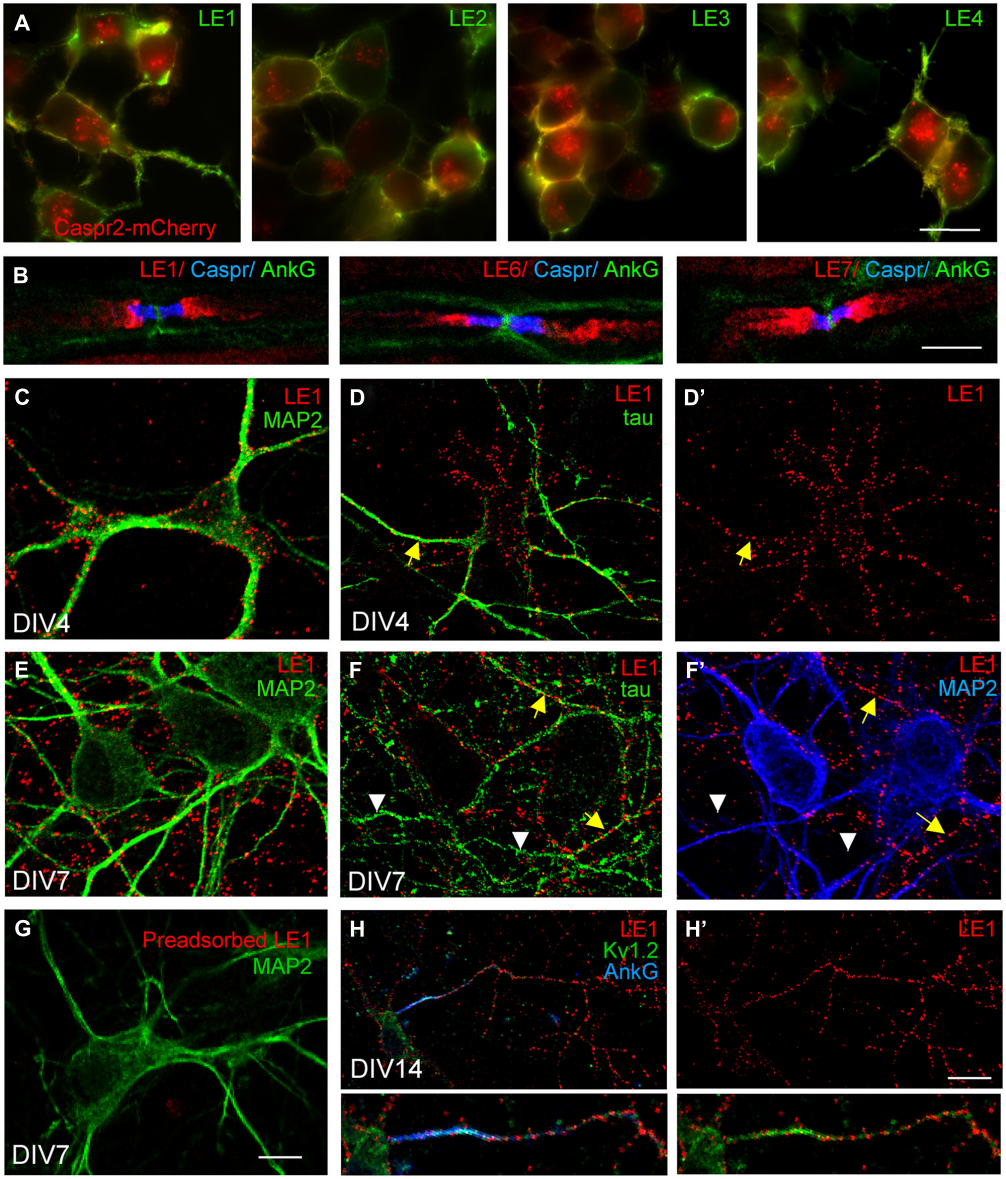
**Limbic encephalitis autoantibodies directed against Caspr2 bind hippocampal neurons in culture. (A)** Neuroblastoma N2a cells were transfected with Caspr2-mcherry (red) and surface labeled with IgGs from LE1–LE4 patients (green). **(B)** Teased sciatic nerve of adult mice were fixed with methanol and immunostained for Contactin-associated protein Caspr (blue) and AnkyrinG (AnkG) (green) as markers of the paranodal and nodal regions of the nodes of Ranvier, respectively. Note that autoantibodies of patients LE1, LE6 and LE7 bound the juxtaparanodes (red). **(C–H)** Rat hippocampal neurons at DIV4 **(C,D,D’)**, DIV7 **(E–G)**, and DIV14 **(H,H’)** were surface labeled with LE1 IgGs (red). Cells were fixed with 4% paraformaldehyde, permeabilized, and double-stained for the somato-dendritic marker MAP2 (green in **C,E,G**; blue in **F’**) or axonal tau (green in **D,F**). Caspr2 surface staining was detected on the somato-dendritic and axonal compartments at DIV4 **(C,D,D’)** but was mainly associated with axonal processes at DIV7 **(E,F,F’)**. In **(F)**, yellow arrows indicate axons that were double-stained for Caspr2 and tau, whereas white arrowheads indicate unlabeled tau-positive axons. **(G)** LE1 IgGs were pre-adsorbed using incubation with Caspr2-transfected HEK cells and did not bind hippocampal neurons. **(H,H’)** Hippocampal neurons at DIV14 were surface labeled for Caspr2 using LE1 IgGs (red) and fixed and permeabilized before immunostaining with mouse-anti-Kv1.2 mAb (green) and rabbit anti-AnkyrinG antibodies (blue). The Kv1.2 channels were enriched at the axonal initial segment stained for AnkyrinG, whereas surface Caspr2 was distributed along the axon. Insets are twofold magnification images. Fluorescence microscopy **(A)** and confocal images **(B–H)**. Bar is in **(A)**, 20 μm; in **(B)**, 5 μm; in **C–G**, 15 μm; in **(H,H’)**, 30 μm.

As shown for patients LE1-4, these autoantibodies strongly labeled Caspr2-transfected N2a cells (**Figure 1A**). Since Caspr2 is a component of the juxtaparanodal VGKC complex, the serum IgGs of patients with LE were tested on teased mouse sciatic nerves. We determined that the serum IgGs of patients bound juxtaparanodes after methanol fixation as shown for LE1, LE6, and LE7 (**Figure 1B**). Next, we showed that the serum IgGs of all patients bound rat hippocampal neurons in culture using live immunostaining (**Figure 1**; **Supplementary Figure S1**). We showed that immunostaining with LE1–LE5 serum IgGs was abolished by pre-adsorption on HEK cells transfected with Caspr2 (**Figure 1G** and **Supplementary Figures S1B,D,F,H**). These data indicate that the IgGs from these patients recognized only Caspr2 in cultured hippocampal neurons.

Previous studies using rabbit anti-Caspr2 antibodies directed against the cytoplasmic region of the molecule, showed that Caspr2 is expressed at low level in axons of cultured hippocampal neurons (Ogawa et al., 2008; Bel et al., 2009) when compared with juxtaparanodes of myelinated axons (Traka et al., 2003). Using anti-Caspr2 LE1 IgGs, we observed surface labeling of the somato-dendritic and axonal compartments of DIV4 neurons double-stained for MAP2 and tau, respectively, (**Figures 1C,D,D**’). At DIV7, a punctate staining of neurites was detected. Double-staining for MAP2 indicated that Caspr2 was faintly expressed at the surface of the somato-dendritic compartment at that stage (**Figures 1E,F,F**’). In contrast, Caspr2 strongly colocalized with the axonal marker tau. Only a subpopulation of axons was immunostained (**Figure 1F**, arrows) whereas some axons were unlabeled (**Figures 1F,F**’, arrowheads). This preferential distribution of Caspr2 at the axonal surface of hippocampal neurons was also observed using live immunostaining with LE2–LE5 serum IgGs at DIV7 (**Supplementary Figures S1A,C,E,G**).

As Caspr2 is known to be associated with Kv1.1/1.2 channels at juxtaparanodes of myelinated fibers and at axon initial segments in various neuronal cell types such as motoneurons or cortical pyramidal cells (Inda et al., 2006; Duflocq et al., 2011), we tested for a possible co-localization in cultured hippocampal neurons. As shown using LE1 serum IgGs in **Figures 1H,H**’ surface staining for Caspr2 was observed along the axon but was not enriched at the axon initial segment, which was strongly stained for Kv1.2 and AnkyrinG at DIV14.

### The N-Terminal Modules of Caspr2 Can be Selectively Targeted by Autoantibodies in LE Patients

Recent studies pointed to the importance of the IgG4 subtype in autoimmune neurological diseases such as myasthenia gravis and CIDP (Huijbers et al., 2013; Labasque et al., 2014; Querol et al., 2014). Using flow cytometry, we studied the IgG specificities of our samples and determined that anti-Caspr2 IgG4 were present in all the LE patients. In addition, IgG4 was the predominant isotype in four out of seven patient’s sera (**Table 2**). The IgG4 do not mediate complement activation, nor bind Fc receptors on effector cells. This could be an indication that the IgG4 in the CSF of LE patients may be pathogenic via functional blocking activity.

**Table 2:** Isotyping and domain mapping of serum and CSF IgGs from LE patients using flow cytometry and cell binding assays.

Patient	Serum		CSF	
	Isotypes	Domain mapping	Isotypes	Domain mapping
LEI	IgGl, IgG2 > IgG3, IgG4	Multiple	IgGl, IgG4	Multiple
LE2	IgGl > IgG4	Multiple	IgGl > IgG4	Multiple
LE3	IgG4	Discoidin-LNGl	IgG4	Discoidin-LNGl
LE4	IgG2 > IgGl, IgG4	Multiple	IgGl	Discoidin-LNGl
LE5	IgG4 > IgGl	Multiple	IgG4 > IgGl	Multiple
LE6	IgG4	Discoidin-LNGl	IgG4	Discoidin-LNGl
LE7	IgG4 > IgGl	Discoidin-LNGl	IgGl, IgG4	Discoidin-LNGl

*HEK cells were transfected with full-length or Δ1-, Δ2-, Δ3-, and Δ4-deleted Caspr2 constructs and were surface-labeled for the HA epitope. Sera at 1:500 dilution were analyzed for IgG1–IgG4 isotypes and CSF at 1:20 dilution for IgG1 and IgG4.*

**FIGURE 2:**
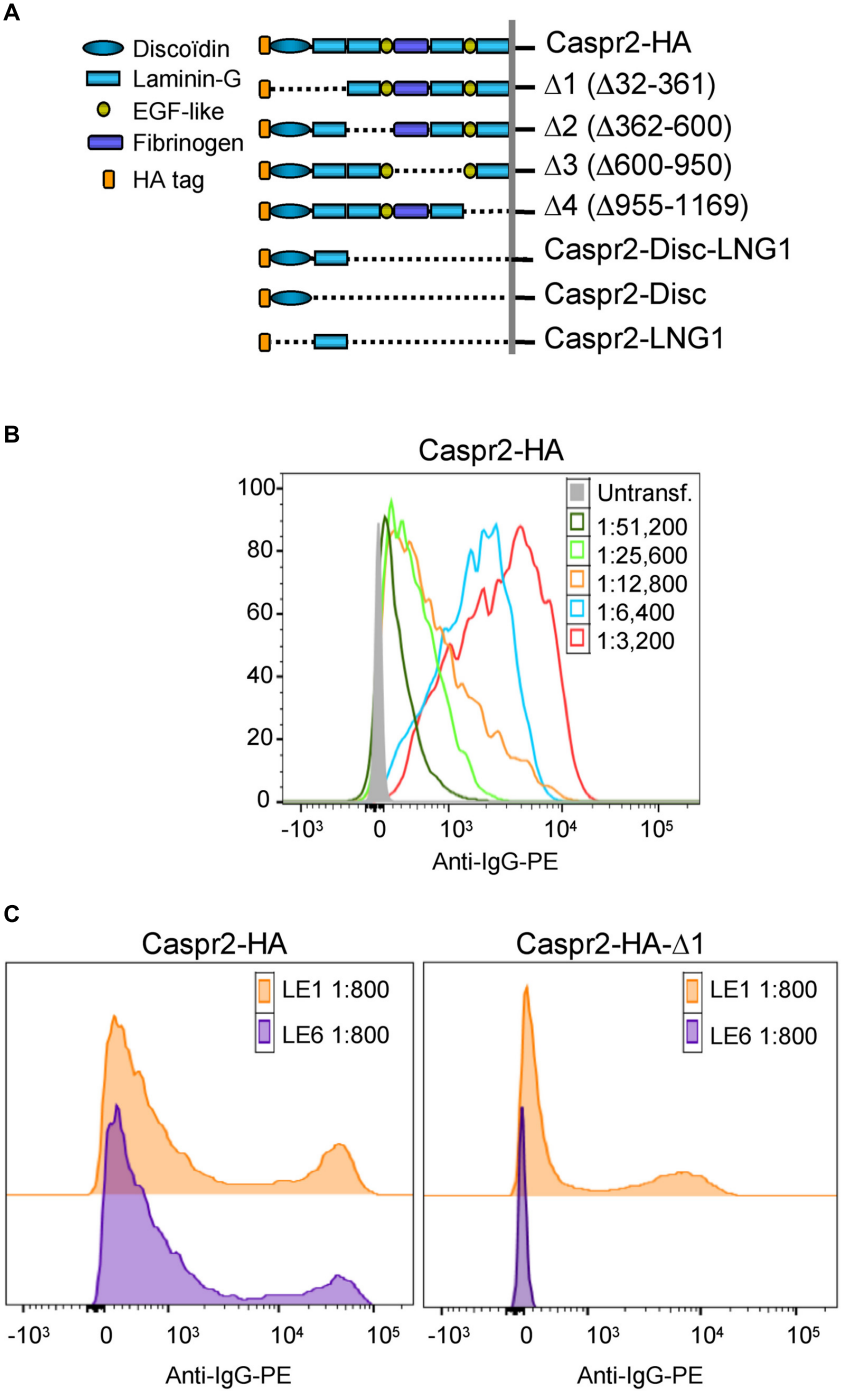
**Epitope mapping of anti-Caspr2 autoantibodies in LE patients. **(A)** Caspr2-HA constructs encompassing sequential deletions: Δ1 deleted of the N-terminal discoïdin and Laminin-G1 domains, Δ2 deleted of the Laminin-G2 and EGF-like1 domains, Δ3 deleted of the central fibrinogen and Laminin-G3 domains, Δ4 deleted of the EGF-like2, and Laminin-G4 domains, Caspr2-Disc-LNG1, Caspr2-Disc, and Caspr2-LNG1 only including the Discoïdin and/or LamininG1 modules in the ectodomain. **(B)** Flow cytometry analysis of anti-Caspr2 IgG titer of LE1 serum. Untransfected and Caspr2-HA-transfected HEK cells were incubated with serial dilutions of LE1 serum (1/3,200–1/51,200) and phycoerythrin-conjugated anti-human IgG (Anti-IgG-PE). **(C)** Flow cytometry analysis of anti-Caspr2 serum IgGs of LE1 and LE6 on HEK cells transfected with full-length or Δ1 deleted Caspr2-HA construct**.

Next, to determine if IgGs of LE patients with anti-Caspr2 autoimmunity may recognize specific modules of Caspr2, we generated Caspr2 constructs encompassing sequential deletions Δ1, Δ2, Δ3, and Δ4 of the protein (**Figure 2A**). Flow cytometry and cell based assays were performed on HEK cells expressing Caspr2 constructs (**Figure 2B**). All patients’ sera recognized the Caspr2-Δ2, Δ3, and Δ4 constructs. On the opposite, three sera and four CSF out of seven did not recognize Caspr2-Δ1 (**Figure 2C**; **Table 2**). Thus, the N-terminal Discoïdin and LamininG1 domains could be a major epitope in patients with LE. To precisely map the epitopes, we generated additional constructs, Caspr2-Discoïdin-LamininG1, Caspr2-Discoïdin, and Caspr2-LamininG1 as depicted in **Figure 2A** and showed that all the seven sera and CSF tested recognized both the Discoïdin and LamininG1 modules, whereas the 30 amino-acids linker between these two modules was not recognized. The specific function of this N-terminal region is still unknown.

### GABAergic Neurons are the Main Target of Anti-Caspr2 Autoantibodies in Patients with LE

**FIGURE 3:**
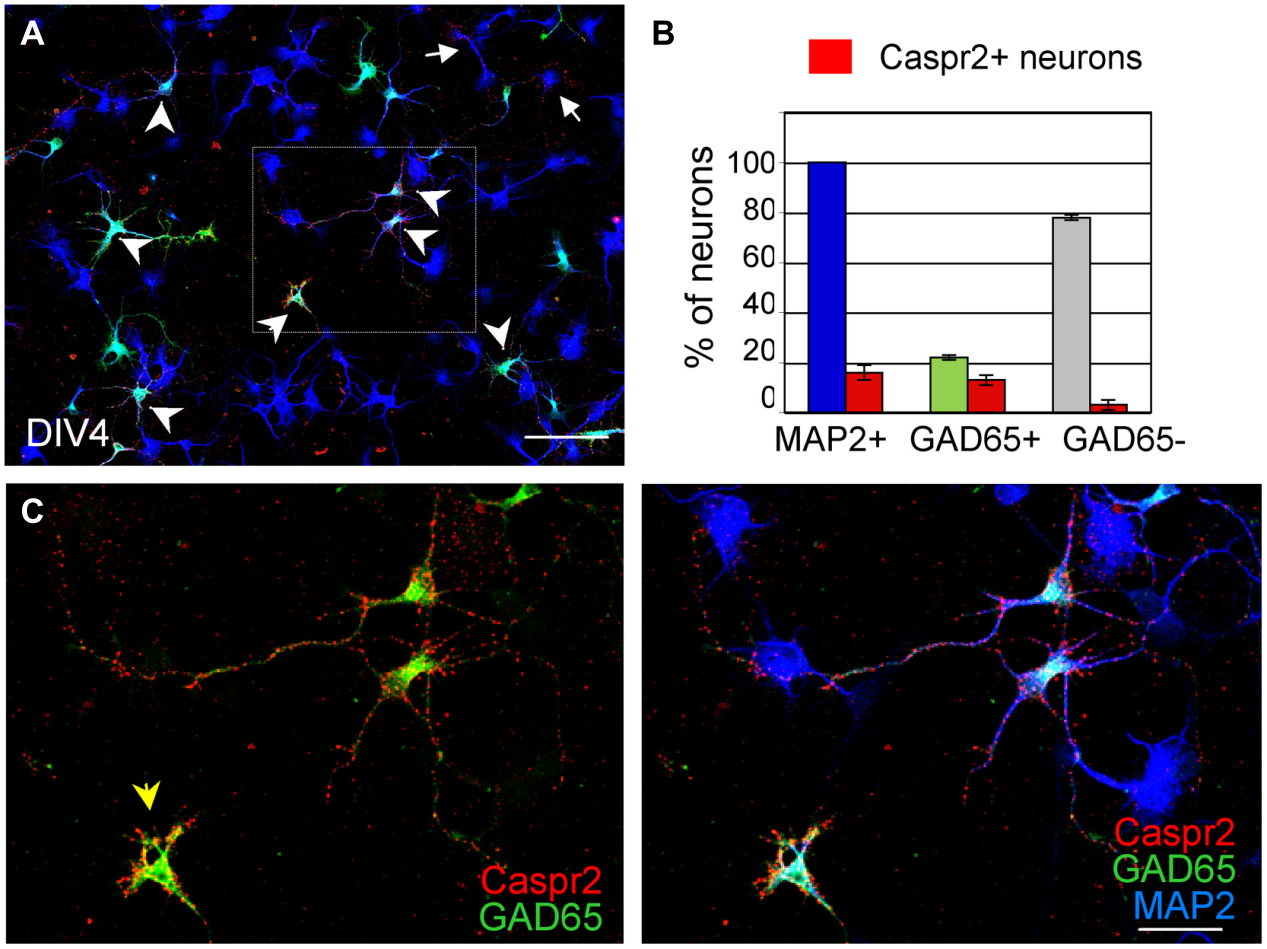
**GAD65-positive neurons are the main target of anti-Caspr2 LE1 autoantibodies.** DIV4 hippocampal neurons were surface labeled with LE1 IgGs (red), fixed and permeabilized before immunostaining for MAP2 (blue) as a neuronal marker and GAD65 (green) to identify inhibitory neurons. **(A)** Low magnification image showing neurons that were surface labeled for Caspr2 (red). Most of them were GAD65-positive (green, arrowheads) and few were GAD65-negative (arrows). The box enlarged in C shows GAD65-positive neurons in green that were surface labeled for Caspr2. The arrowhead points to an isolated inhibitory neuron strongly labeled for Caspr2 on the soma **(B)** Quantitative analysis of the percentage of total neurons, GAD65-positive and GAD65-negative neurons that were surface labeled for Caspr2. Means ± SEM of three independent experiments. **(A)** Tiling of 5 × 4 confocal images acquired with the 63x objective, *z*-stack of 6 confocal sections with *z*-step of 0.5 μm. Bar is in A, 80 μm; in **(C)**, 35 μm.

Since only a subpopulation of axons was labeled using anti-Caspr2 LE1 serum IgGs, we further investigated whether excitatory or inhibitory subpopulation of neurons may be differentially targeted. DIV4 neurons were analyzed to study the somato-dendritic expression of Caspr2 at early stage. We first determined that 22% of MAP2-positive neurons were GABAergic using glutamate decarboxylase GAD65 as a marker (**Figures 3A,B**). Next using immunolabeling with LE1 serum IgGs, we estimated that 58% of the inhibitory neurons in contrast to only 4% of excitatory neurons (negative for GAD65, arrows) expressed Caspr2 at DIV4 (**Figures 3A,B**; **Table 3**). Thus, most of the Caspr2-positive neurons (81%) were inhibitory neurons as illustrated in **Figure 3C**. In the same manner, quantitative analyses were performed using LE2–LE5 serum IgGs and indicated that 51–68% of GAD65-positive neurons and only 4–8% of GAD65-negative neurons were targeted by these patients IgGs (**Table 3**).

**Table 3:** The anti-Caspr2 autoantibodies in LE patients target inhibitory neurons.

			% Caspr2-positive neurons		
	*(n)*	% inhibitory neurons GAD65-positive	Total MAP2-positive	Inhibitory GAD65-positive	Excitatory GAD65-negative
LEI	434	22 ± 1	16 ± 3	58 ± 5	4 ± 2
LE2	480	17.4 ± 4	15 ± 3.8	51.2 ± 3.7	7.2 ± 3.7
LE3	374	23.9 ± 1	18.9 ± 4.5	57.4 ± 8.9	6.7 ± 3.2
LE4	354	19.9 ± 3.5	19.1 ± 3.9	64.3 ± 4.8	8.3 ± 3.3
LE5	358	21.5 ± 1.2	19.9 ± 2.7	68.5 ± 8.2	6.5 ± 1.2

*After four days *in vitro* hippocampal neurons were surface labeled with LE1–LE5 IgGs and double-stained for MAP2 as a neuronal marker and GAD65 to identify inhibitory neurons. (*n*) indicates the total number of neurons analyzed on three different coverslips.*

**FIGURE 4:**
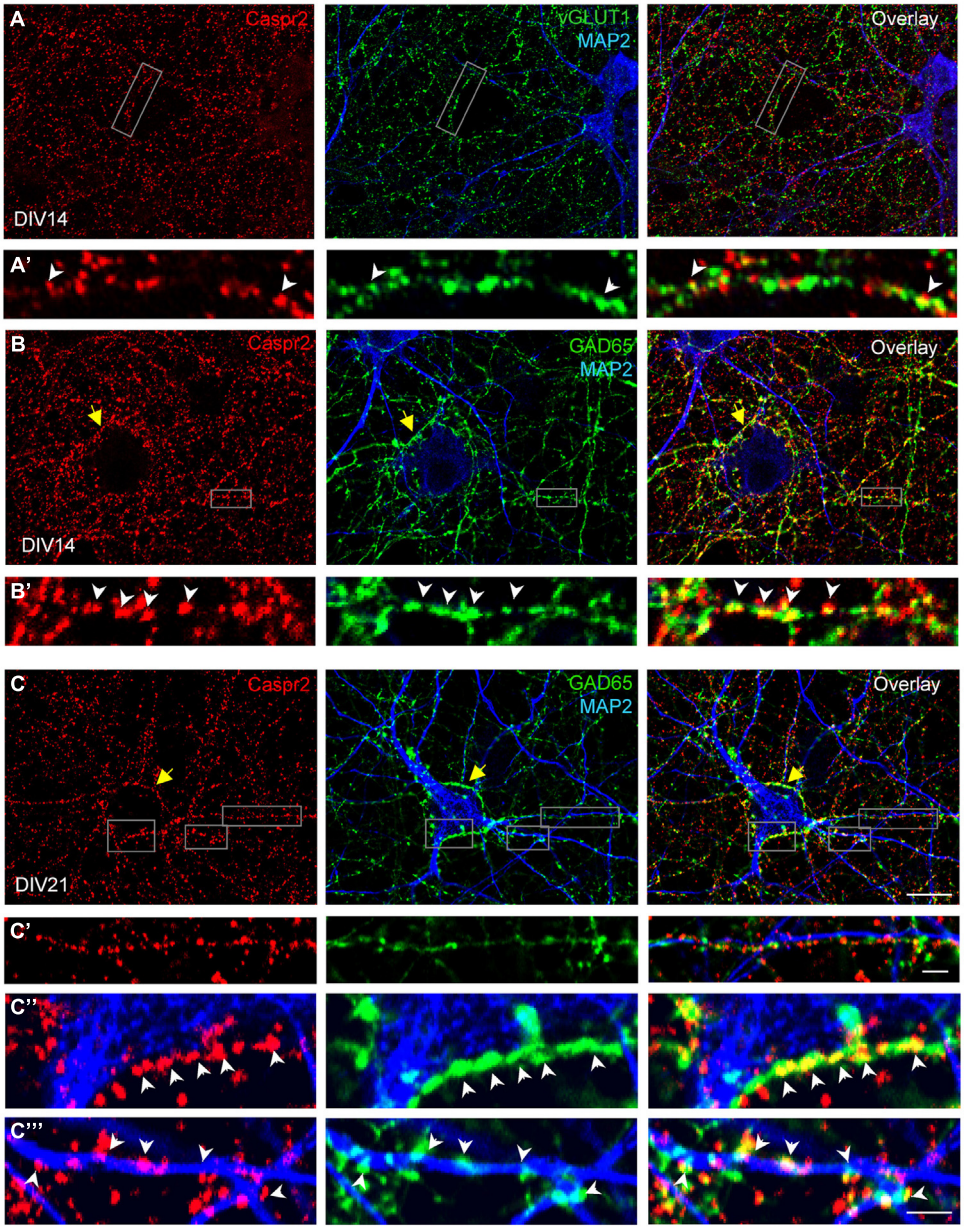
**Distribution of Caspr2 at pre-synaptic sites of excitatory and inhibitory axons.** Confocal images of hippocampal neurons at DIV14 **(A,B)** or DIV21 **(C)** that were surface labeled for Caspr2 using LE1 IgGs (red). Cells were fixed and permeabilized before double-staining for MAP2 (blue), vGLUT1 (**A**, green) or GAD65 (**B,C**, green). **(A’)** Enlarged areas shows glutamatergic pre-synaptic sites stained for Caspr2. **(B’,C’–C”’)** Inhibitory pre-synaptic sites labeled for GAD65 (arrowheads) were intensely stained for Caspr2 at the contact with the soma **(C”)** or dendrites **(B’,C”’)**. Note that GAD65-positive axons surrounding the soma of pyramidal neurons were heavily stained for Caspr2 (yellow arrows). **(C’)** This enlarged area shows Caspr2 punctate immunostaining along a GAD65-positive axon apposed to a dendrite. Bar is in **(A–C)**, 9 μm; in insets, 1.5 μm.

We also examined how Caspr2 was distributed along axons and pre-synaptic sites at DIV14 and DIV21 later stages. The vesicular glutamate transporter-1 vGLUT1 was used as a marker for glutamatergic axons and synapses. As shown in **Figure 4A**, some of the vGLUT1-positive axons at DIV14 were labeled for Caspr2 using LE1 serum IgGs. High magnification images show that Caspr2 co-localized with vGLUT1 at pre-synaptic sites (**Figures 4A**’, arrowheads). Next we used GAD65, which synthesizes GABA for neurotransmission as a marker for inhibitory axons and synapses. Caspr2 strongly co-localized with GAD65-positive axons as observed using serum IgGs of all the patients analyzed (**Supplementary Figure S2**). In particular, the GAD65-positive axons were surrounding the MAP2-labeled large pyramidal neurons and were heavily stained for Caspr2 at DIV14 and DIV21 as shown using LE1 serum IgGs (arrows in **Figures 4B,C**). The presynaptic sites labeled for GAD65 were intensely stained for Caspr2 at the contact with the soma (arrowheads in **Figures 4C”**) or dendrites (arrowheads in **Figures 4B’,C”’**). We estimated that 51% of the GABAergic pre-synaptic contacts on dendrites were labeled for Caspr2 (5.9 ± 0.5 Caspr2-positive of 11.6 ± 0.5 total GABAergic contacts/25 μm dendritic length; *n* = 14 dendrites). As illustrated in **Figures 4C**’, Caspr2 was distributed along inhibitory axons partially overlapping with GAD65 puncta. We estimated that 34 % of the Caspr2-positive clusters contacting dendrites were GAD65-positive (13.7 ± 2 Caspr2-positive clusters among which 4.7 ± 0.8 were GABAergic/25 μm).

**FIGURE 5:**
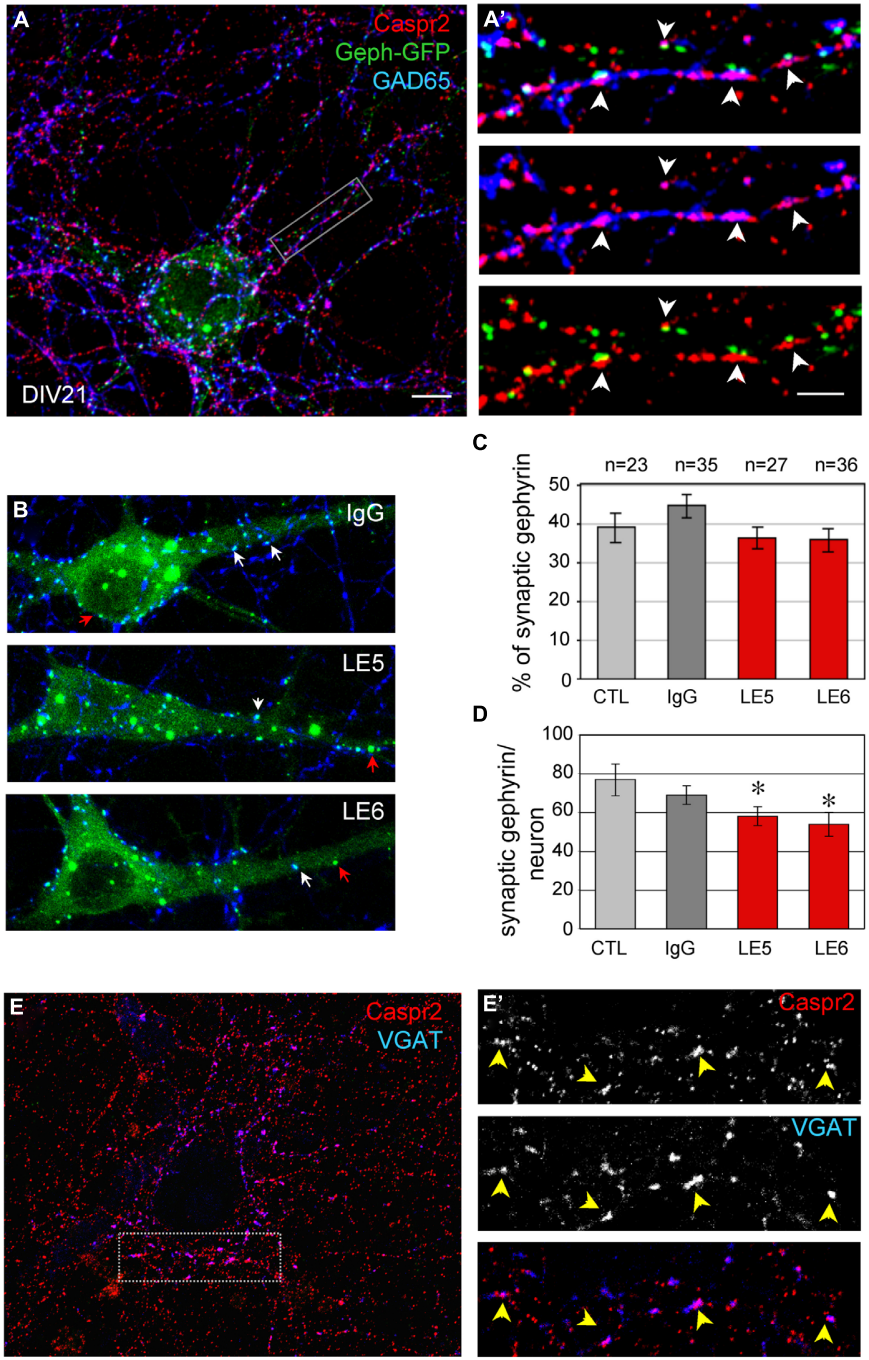
**(A)** Neurons were transfected with Gephyrin-GFP (Geph-GFP, green) at DIV14 and labeled at DIV21 for surface Caspr2 (red) and GAD65 (blue). **(A’)** The insets show the pre-synaptic sites double-labeled for Caspr2 and GAD65 facing post-synaptic clusters of Gephyrin-GFP (arrowheads). **(B)** Hippocampal neurons were transfected with Gephyrin-GFP (green) at DIV14 and incubated at DIV17 with control, LE5 or LE6 IgGs (1/100 dilution) for 1 h at 37°C. Clusters of post-synaptic Gephyrin in contact with presynaptic GAD65 are indicated with white arrows and non-synaptic Gephyrin with red arrows. **(C)** Quantitative analysis of the ratio of synaptic relative to total Gephyrin clusters under the different experimental conditions: incubation with culture medium (CTL), or with control, LE5 or LE6 IgGs. **(D)** Number of synaptic Gephyrin-GFP clusters per neuron. Means ± SEM, n indicates the number of neurons analyzed. ^∗^indicates significant difference (*P* < 0.05) with the culture medium condition using ANOVA followed by Fischer’s test. **(E)** Double-staining for surface Caspr2 (red) and VGAT as a marker of inhibitory pre-synaptic terminals with the box enlarged in **(E’)**. Note the colocalisation of Caspr2 and VGAT indicated with arrowheads. **(A,A’, B,E)**: *Z*-stack of five confocal sections with *z*-step of 0.5 μm. **(E’)** is a single confocal section. Bar is in **(A,B,E)**, 10 μm; in insets **(A’,E’)**, 1.5 μm.

Hippocampal neurons were transfected at DIV14 with Gephyrin-GFP to visualize the post-synaptic clusters facing pre-synaptic inhibitory contacts at DIV21 (**Figure 5A**). Gephyrin is the major post-synaptic scaffolding protein at inhibitory synapses (Craig et al., 1996). As shown in **Figures 5A**’, pre-synaptic terminals positive for both GAD65 and Caspr2 were apposed to Gephyrin-GFP clusters (arrowheads). In addition, surface Caspr2 colocalized with the inhibitory presynaptic terminals that were labeled for VGAT (**Figures 5E,E**’). We asked whether the LE patient’s autoantibodies directed against Caspr2 could display functional blocking activity by destabilizing inhibitory synaptic contacts. The LE5 and LE6 autoantibodies were tested which are mainly of the IgG4 isotype. LE5 IgGs are directed against multiple domains of Caspr2 and LE6 IgGs only target the N-terminal modules. Hippocampal neurons were transfected with Gephyrin-GFP at DIV14 and incubated at DIV17 with the culture medium, control IgGs, LE5, or LE6 IgGs diluted 1:100 for 1 h at 37°C. Using automatic spot detection of the Imaris software, we determined under each condition the number of total Gephyrin-GFP clusters and the number of Gephyrin-GFP clusters apposed to GAD65-positive presynaptic terminals (**Figure 5B**, white arrows). The ratio of synaptic versus total Gephyrin-GFP clusters was not significantly affected by incubation during 1 h with LE autoantibodies (**Figure 5C**). However, a significant decrease in the number of synaptic Gephyrin clusters per neuron was observed for LE5 and LE6 (24.5 and 30%, respectively, *P* < 0.05 using ANOVA and Fisher’s test) but not for control IgGs (10%) by comparison with culture medium incubation (**Figure 5D**). The number of GAD65-positive clusters contacting the somato-dendritic compartment per Gephyrin-GFP transfected neuron was not significantly decreased (284 ± 41 with culture medium, 230 ± 18 with control IgGs, 218 ± 21 with LE5, 210 ± 30 with LE6). Since Caspr2 was only expressed in 60% of GAD65-positive neurons, the functional effect of LE autoantibodies may be underestimated. In conclusion, we observed that Caspr2 is selectively localized along GABAergic axons and at the inhibitory pre-synaptic terminals in cultured hippocampal neurons. In addition, the perturbating assays of post-synaptic Gephyrin clusters suggest that anti-Caspr2 autoantibodies of LE patients may be pathogenic by altering the inhibitory synaptic contacts.

### The Caspr2-Fc Binding Sites are Localized on the Somato-Dendritic Compartment

**FIGURE 6:**
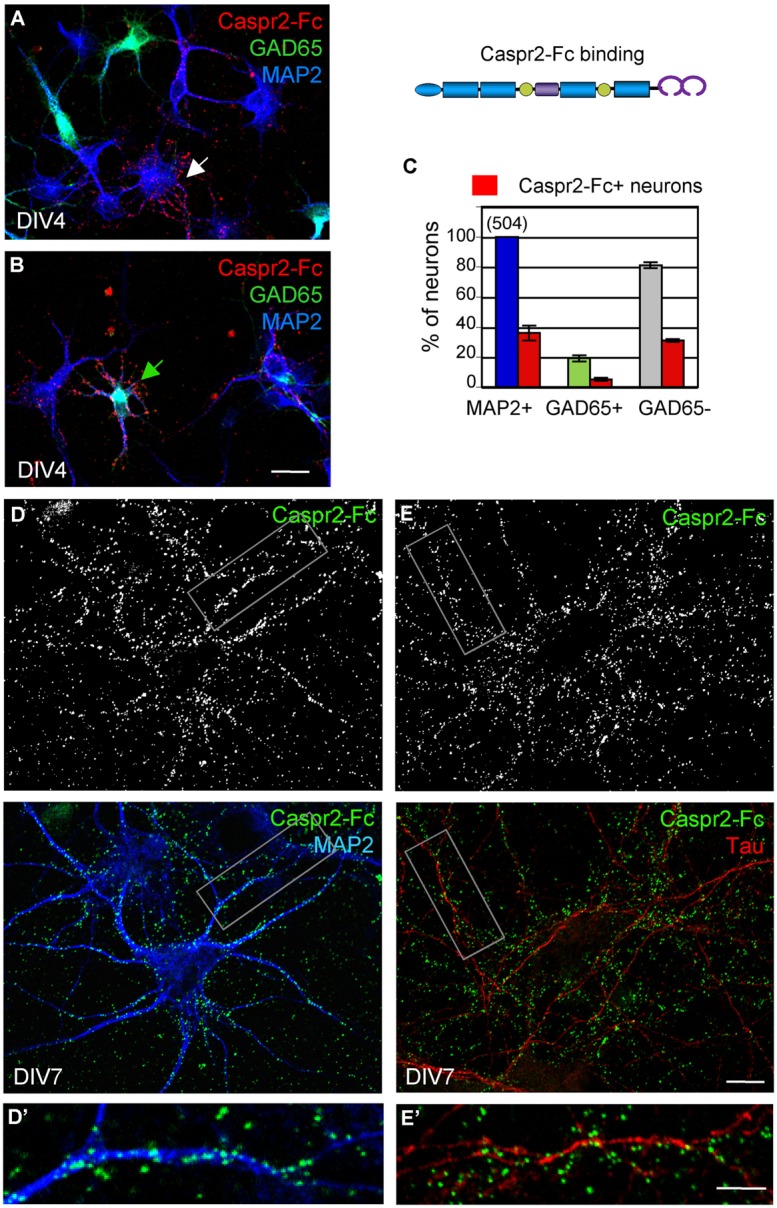
**The binding sites of Caspr2-Fc are localized on the somato-dendritic compartment.** Hippocampal neurons at DIV4 **(A–C)** and DIV7 **(D,E)** were incubated with 10 μg/ml Caspr2-Fc preclustered with Alexa-conjugated anti-Fc IgGs for 30 min at 37°C. **(A,B)** DIV4 neurons bound with Caspr2-Fc (red). Cells were fixed and permeabilized before double-staining for MAP2 (blue) and GAD65 (green). **(A,B)** show representative images of GAD65-negative neurons (white arrow, **A**) and GAD65-positive neurons (green arrow, **B**) labeled with Caspr2-Fc. **(C)** Quantitative analysis of the percentage of total neurons, GAD65-positive and GAD65-negative neurons that were surface labeled for Caspr2-Fc. Means ± SEM of three independent experiments, *n* = 504 neurons. **(D,E)** DIV7 neurons bound with Caspr2-Fc (green) were double-stained for MAP2 (**D**, blue) or tau (**E**, red). The insets in **(D’,E’)** show that Caspr2-Fc preferentially bound on MAP2-positive dendrites and not on tau-positive axons. **(A,B)** *z*-stacks of six confocal sections with *z*-step of 0.5 μm. **(D,E)** Single optical sections of confocal images. Bar is in **(A,B)**, 20 μm; in **(D,E)**, 10 μm; in insets, 7 μm.

Caspr2 belongs to the family of neurexins, which are pre-synaptic CAMs. Studies in culture indicate that neurexins are implicated in synaptogenesis by inducing the clustering of post-synaptic neuroligins (Dean et al., 2003; Craig and Kang, 2007). Neurexin/neuroligin association promotes the formation of excitatory and inhibitory synapses by interacting with PSD95 or Gephyrin, respectively. We asked whether Caspr2 might be also involved in trans-synaptic contacts. With this aim, we generated a Caspr2-Fc chimera to detect Caspr2 binding sites in hippocampal neuronal culture. Caspr2-Fc plasmid was transfected in HEK cells and the recombinant protein purified from the culture supernatant using Protein A-affinity chromatography. The chimera pre-clustered with fluorescent anti-Fc IgG was incubated with hippocampal neurons at DIV4 (**Figure 6**). We observed that Caspr2 binding sites were present on both GAD65-negative (arrow in **Figure 6A**) and GAD65-positive neurons (green arrow in **Figure 6B**). Quantitative analysis indicated that Caspr2-Fc bound 36% of the total neurons (**Figure 6C**). Caspr2-Fc bound to the somato-dendritic compartment of DIV7 neurons as determined using double-staining for MAP2 (**Figures 6D,D**’). In contrast, Caspr2-Fc was not co-localized with axons immunostained with anti-tau mAb (**Figures 6E,E**’). Caspr2-Fc binding sites were distributed on the somato-dendritic compartment of both inhibitory (26 ± 6%) and excitatory (38 ± 5%) neurons as analyzed at DIV4 (*n* = 504 neurons, three coverslips; **Figure 6C**).

**FIGURE 7:**
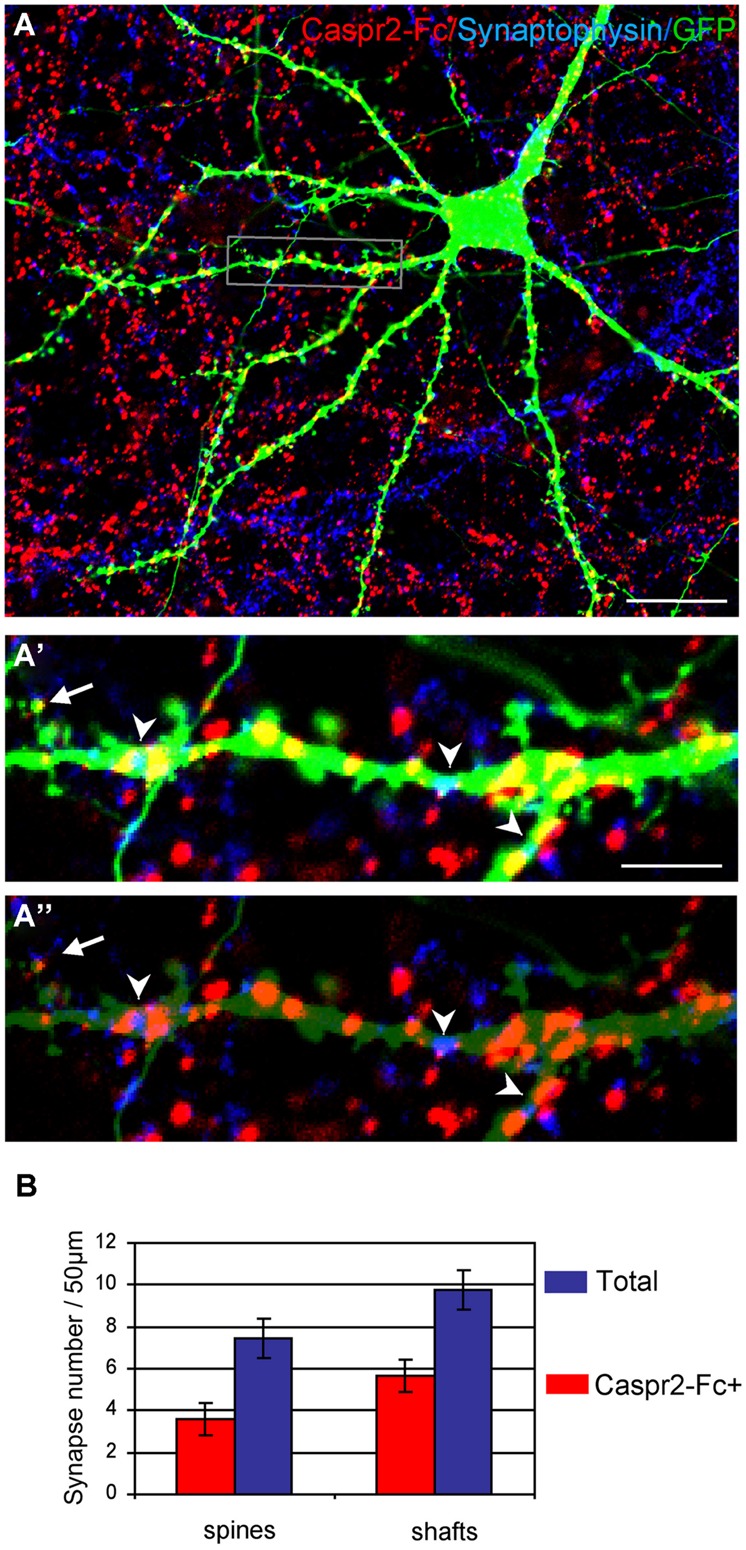
**The Caspr2-Fc binding sites are localized at post-synaptic contacts. (A)** Hippocampal neurons were transfected at DIV14 with GFP and incubated at DIV21 with preclustered Caspr2-Fc (red). Cells were fixed, permeabilized and immunostained for Synaptophysin (blue). **(A’,A”)** Insets show the distribution of Caspr2-Fc binding sites (red) on the dendritic shaft (arrowheads) or on spines (arrow) that were contacting Synaptophysin pre-synaptic sites (blue). The green channel is turned down in **(A”)** to visualize Synaptophysin clusters. **(B)** Quantitative analysis of the number of post-synaptic sites bound with Caspr2-Fc on spines and shaft. Means ± SEM, *n* = 21 dendrites of seven neurons. Single confocal sections. Bar is in **(A,B)**, 20 μm; in insets, 4 μm.

Next, we analyzed whether the Caspr2 binding sites may be distributed at the post-synaptic sites. Neurons were transfected with GFP at DIV14 to clearly visualize dendrites and spines of isolated neurons at DIV21 (**Figure 7A**). Synaptic contacts on dendritic shafts (arrowheads) or spines (arrow) were detected using Synaptophysin as a pre-synaptic marker. As shown in high magnification pictures, Caspr2 binding sites (red) were detected on shafts at the contact with the pre-synaptic marker (blue; **Figures 7A’,A”**). Quantitative analysis indicated that 58% of post-synapses on shafts and 48% on spines were labeled with Caspr2-Fc chimera (*n* = 21 images; **Figure 7B**). Thus, the receptors of Caspr2 were present both at inhibitory and excitatory post-synaptic sites on shafts and spines, respectively. The Caspr2-binding sites at the inhibitory post-synapses were complementary to the distribution of Caspr2 at inhibitory pre-synaptic sites.

### The Somato-Dendritic Binding Sites of Caspr2 Depends on TAG-1 but not on LGI1, ADAM22, and ADAM23

**FIGURE 8:**
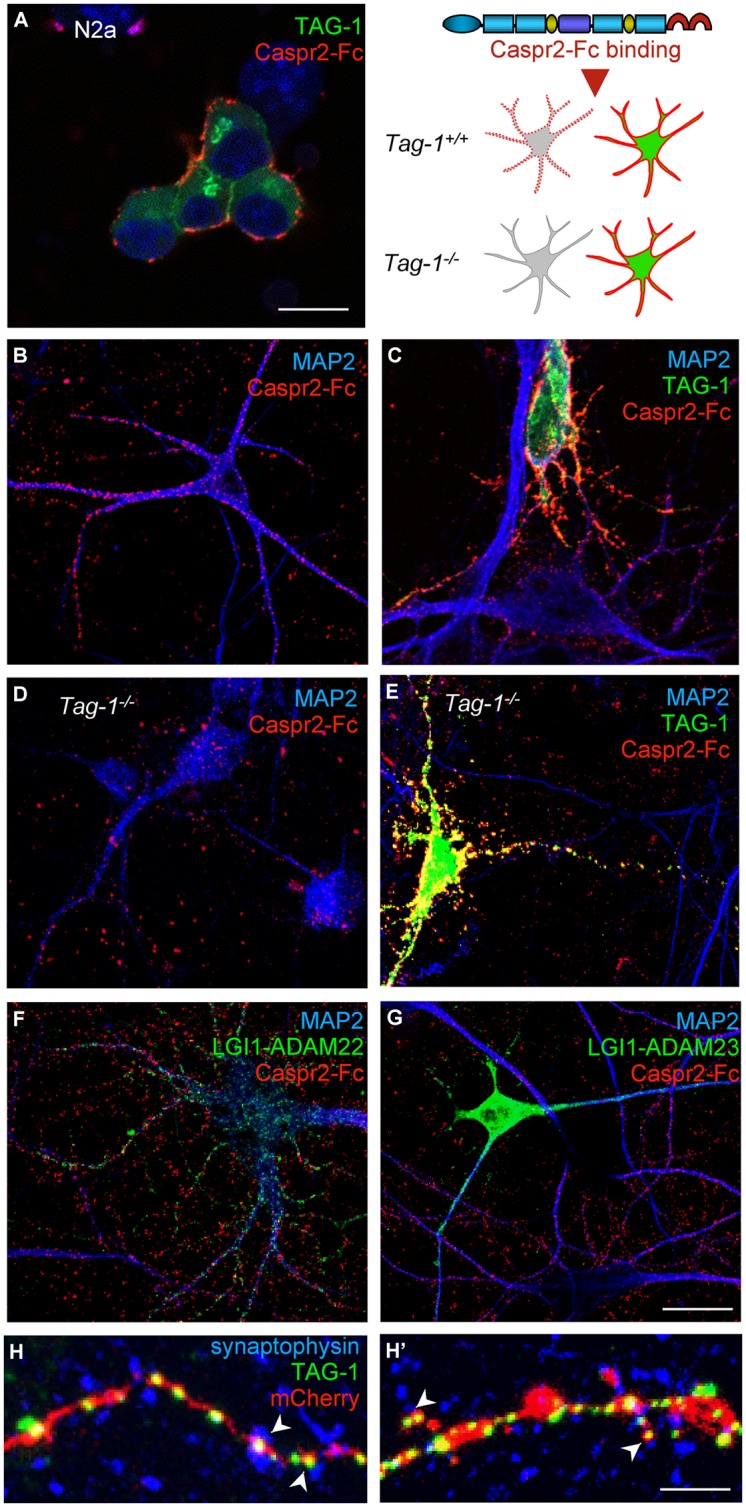
**TAG-1 is required for Caspr2-Fc binding on hippocampal neurons. (A)** N2a neuroblastoma cells were transfected with TAG-1-GFP (green) and incubated with preclustered Caspr2-Fc (red). Caspr2-Fc only bound on TAG-1-GFP expressing N2a cells. **(B–G)** DIV8 hippocampal neurons were incubated with pre-clustered Caspr2-Fc (red) and cells were fixed and permeabilized before immunostaining for MAP2 (blue). Wild-type neurons were untransfected **(B)** or transfected with TAG-1-GFP **(C)**, or double-transfected with LGI1-GFP and ADAM22 **(F)** or LGI1-GFP and ADAM23 **(G)**. Caspr2-Fc strongly bound the TAG-1-GFP-expressing neuron (green) by comparison with untransfected neurons **(C)**. Transfection of LGI-GFP and ADAM22 or ADAM23 had no effect. **(D,E)** DIV8 hippocampal neurons from *Tag-1^-/-^* mice were untransfected **(D)** or transfected with TAG-1-GFP **(E)**. Caspr2-Fc strongly bound the TAG-1-GFP-expressing neuron (green) and did not bind untransfected *Tag1^-/-^* neurons. **(H,H’)** DIV14 hippocampal neurons were co-transfected with TAG-1-GFP and mCherry. At DIV17, neurons were surface labeled with anti-GFP antibodies, fixed, and permeabilized before immunostaining for Synaptophysin (blue). Note that TAG-1-GFP clusters indicated with arrowheads on the shaft **(H)** or spines **(H’)** were facing Synaptophysin presynaptic sites. **(A–G)** Single confocal sections. **(H,H’)** z-stacks of four confocal sections with *z*-step of 0.5 μm Bars: 10 μm, in **(A–G)**, 4 μm in **(H,H’)**.

Contactin 2/TAG-1 is an Ig-CAM that associates with Caspr2 and the VGKC complex at juxtaparanodes (Poliak et al., 2003; Traka et al., 2003). It was previously reported that TAG-1 strongly associates in cis with Caspr2 whereas the trans-interaction remains elusive, since TAG-1-Fc does not bind to Caspr2 expressed at the cell membrane of HEK cells (Traka et al., 2003). Conversely, we observed that the pre-clustered Caspr2-Fc chimera bound N2a cells transfected with GPI-anchored TAG-1 fused with GFP downstream the signal peptide (**Figure 8A**). In addition, Caspr2-Fc binding was strongly enhanced on neurons transfected with TAG-1 when compared with untransfected neurons (**Figures 8B,C**), indicating that the trans-interaction with TAG-1 strongly occurs in neuronal cells. Next, we tested whether Caspr2-Fc bound on hippocampal neurons from *Tag-1^-/-^* mice. Caspr2-Fc binding was faintly detected on TAG-1-deficient neurons (**Figure 8D**). In contrast, Caspr2-Fc strongly labeled hippocampal neurons from *Tag-1^-/-^* mice that were transfected with TAG-1-GFP in contrast with untransfected neurons (**Figure 8E**).

We also tested whether the binding of Caspr2-Fc may depend on LGI1/ADAM22 that are members of the VGKC complex and are recruited at the post-synapse (Fukata et al., 2006; Owuor et al., 2009; Ogawa et al., 2010). We did not observe any binding of Caspr2-Fc on N2a cells transfected with LGI1 alone or co-transfected with LGI1 and ADAM22 or ADAM23 (not shown). In addition, Caspr2-Fc binding was not increased on hippocampal neurons transfected with LGI1 and ADAM22 or ADAM23 by comparison with untransfected neurons (**Figures 8F,G**).

To analyze the subcellular targeting of transfected TAG-1, neurons were double-transfected with TAG-1-GFP and mCherry to visualize the dendritic arborisation (**Figures 8H,H**’). Using live immunostaining with anti-GFP antibodies, we observed that TAG-1 was addressed to the surface of dendritic arborisation including at the dendritic spines at DIV17. TAG-1-GFP clusters were facing presynaptic sites labeled for synaptophysin both on shafts and spines (**Figures 8H,H**’, respectively). These data indicated that transfected TAG-1 was detected post-synaptically as observed for Caspr2-Fc binding sites. We concluded that TAG-1 may be critically required for Caspr2-Fc binding on the post-synaptic compartment of hippocampal neurons.

## Discussion

**FIGURE 9:**
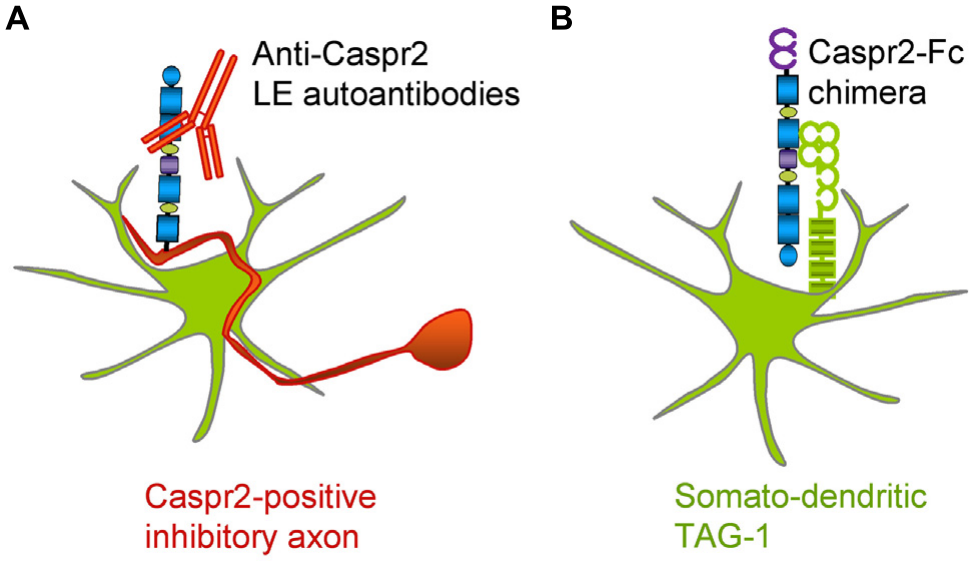
**Schematic representation of Caspr2 and Caspr2-Fc complementary distribution in hippocampal neurons. (A)** Anti-Caspr2 autoantibodies target preferentially the axons of inhibitory neurons. **(B)** TAG-1 is required as a post-synaptic receptor of Caspr2.

In the present study, we analyzed autoantibodies against Caspr2 in a series of patients with LE. First, we determined that IgGs in the CSF of four out seven patients selectively react against the Discoïdin and LamininG1 N-terminal modules of Caspr2. Second, using live staining of hippocampal neurons in culture, we showed that autoimmunity to Caspr2 mainly targets hippocampal inhibitory interneurons (**Figure 9A**). Anti-Caspr2 IgGs label GAD65-positive pre-synaptic sites apposed to Gephyrin post-synaptic clusters. Functional assays indicated that LE autoantibodies may induce alteration of inhibitory synaptic contacts. Third, we used a Caspr2-Fc chimera to reveal Caspr2 receptors on hippocampal neurons. Caspr2 binding sites are distributed on the somato-dendritic compartment at post-synaptic sites. We showed that TAG-1 expression is essential for Caspr2-Fc binding on hippocampal neurons (**Figure 9B**). These results indicate that Caspr2 may participate as a cell recognition molecule in the dynamics of inhibitory networks. In addition, they point out to the immune targeting of inhibitory synapses as a critical clue for understanding the physiopathological role of Caspr2.

### Distinct Axonal Distributions of Caspr2 and Kv1 Channels in Hippocampal Neurons

We previously reported that transfected Caspr2 is preferentially addressed to the axon and strongly internalized in the somato-dendritic compartment in cultured hippocampal neurons at DIV7 (Bel et al., 2009). The surface expression of Caspr2 is controlled via a Protein Kinase C-regulated motif of endocytosis in its cytoplasmic tail (Bel et al., 2009) that might also interfere with availability of the VGKC-complexes. In the present study, we used anti-Caspr2 IgGs of LE patients, which display very high titer, to label endogenous Caspr2. We showed that Caspr2 is preferentially expressed at the axonal surface, but not enriched at the axon initial segment as observed for Kv1 channels and contactin 2/TAG-1 at DIV14. The Kv1.1/1.2 channels display axonal distribution and are tethered at the axon initial segments in pyramidal cortical neurons and also in inhibitory neurons in hippocampal cell culture and slices (Sanchez-Ponce et al., 2012; Campanac et al., 2013). The recruitment of Caspr2 at the initial segment might be regulated or its antigenicity masked when associated with the VGKC complex. However, we showed that anti-Caspr2 IgGs of several patients bound to VGKC complex at juxtaparanodes of myelinated axons in culture and were reactive for dendrotoxin-precipitated VGKC.

### Caspr2 is a Pre-Synaptic CAM Trans-Interacting with TAG-1

Caspr2 functional role may be partly independent from its association with the VGKC-complexes. Indeed, Caspr2 may participate in cell adhesive processes and play a morphogenetic role in neurite outgrowth and synaptogenesis (Anderson et al., 2012). The phenotypic analysis of Caspr2 knockout mice points to its involvement in the migration of cortical neurons and generation or positioning of interneurons (Penagarikano et al., 2011). The number of parvalbumin-positive interneurons is reduced in Caspr2 mutant mice associated with decreased synchronous firing of cortical neurons. However, evidence for synaptic alterations has not been reported. Alteration of developmental events may underlie the spontaneous seizures and behavioral disorders observed in adult Caspr2 knockout mice (Penagarikano et al., 2011).

Our data suggest a cell adhesive or cell-recognition function of pre-synaptic Caspr2 interacting with TAG-1 at the post-synaptic compartment. TAG-1 is the single interacting partner already identified for Caspr2 ectodomain, and their cis-association has been clearly evidenced (Poliak et al., 2003; Traka et al., 2003). We showed here that pre-clustered Caspr2-Fc binds TAG-1 anchored at the membrane of N2a cells indicating *trans*-interaction between the two CAMs. In contrast, the reciprocal interaction is not occurring between soluble TAG-1-Fc and Caspr2-expressed at the cell membrane (Traka et al., 2003). Our recent data indicate that the two molecules expressed at the membrane of opposing cells can mediate trans-adhesive interaction as determined by co-immunoprecipitation (Savvaki et al., 2010).

Both Caspr2 and TAG-1 are present in the fraction containing synaptic plasma membranes (Bakkaloglu et al., 2008) with Caspr2 highly depleted in the post-synaptic density fraction (Chen et al., 2015). TAG-1 is faintly expressed at the surface of cultured hippocampal neurons and detected at axon initial segment (Ogawa et al., 2008) and it is also released as a soluble form (Karagogeos et al., 1991). Strikingly, we observed that Caspr2-Fc binds the somato-dendritic compartment of wild-type but not of TAG-1-deficient hippocampal neurons. We showed that Caspr2-Fc binding was strongly increased on TAG-1-transfected neurons. LGI1 was also considered as a possible post-synaptic receptor for Caspr2 as it is recruited within the VGKC complex where it interacts with ADAM22 and ADAM23 (Ogawa et al., 2008) and LGI1 is also involved in some autoimmune encephalitis (Lai et al., 2010). However, we did not detect any trans-interaction of Caspr2-Fc with any of these components. We showed that both Caspr2-Fc binding sites and transfected TAG-1-GFP were localized at post-synaptic sites on dendritic shafts and spines facing synaptophysin-positive clusters. Thus, taken together these data suggest that TAG-1 may be critically involved as a post-synaptic partner of Caspr2. We observed that half of excitatory post-synapses on spines were labeled with Caspr2-Fc chimera, whereas Caspr2 was mainly expressed by inhibitory neurons. Indeed, TAG-1 has been reported to interact with several members of the L1 family (Felsenfeld et al., 1994; Pavlou et al., 2002) and may interplay with other partners at the excitatory synapse.

### Caspr2 and LGI1 may be Differentially Implicated in LE Autoimmunity

Both LGI1 and Caspr2 are targeted in some patients with autoimmune LE (Irani et al., 2010; Lai et al., 2010). LGI1 is also implicated in inherited forms of epilepsy (Morante-Redolat et al., 2002). Like Caspr2, LGI1 is an element of the VGKC-complex and may induce alteration of axonal excitability. LGI1 colocalizes at axonal terminals with Kv1.1 and Kv1.4 and is strongly expressed on mossy fibers in the hippocampus (Schulte et al., 2006). Interestingly, anti-LGI1 autoantibodies of patients with LE have been reported to induce epileptiform activity by increasing the release probability on mossy fibers-CA3 pyramidal cell synapses, an effect that is mimicked by antagonists of Kv1 channels (Lalic et al., 2010). Other works demonstrated that LGI1 associated with ADAM22 is also implicated in regulating synaptic transmission (Fukata et al., 2006) and that anti-LGI1 autoantibodies of patients with LE are able to neutralize LGI1-ADAM22 interaction and to reduce synaptic clusters of AMPA receptors in cultured hippocampal neurons (Ohkawa et al., 2013). Since LGI1 is expressed by inhibitory interneurons as well as excitatory neurons in hippocampus, epileptic activity may be induced by function blocking of LGI1 on interneurons. However, the selective deletion of *Lgi1* in GABAergic parvalbumin neurons does not induce spontaneous seizures or increased seizure susceptibility. In contrast, depletion of LGI1 in pyramidal neurons is sufficient to generate seizures suggesting that LGI1 plays a pathological role in specific neurons (Boillot et al., 2014). Thus, it may be important to identify the subtypes of neurons expressing Caspr2 to decipher the role of this CAM in LE.

In the present study, we showed that Caspr2 is strongly expressed by inhibitory neurons in hippocampal cell culture including at their presynaptic terminals. Gephyrin is a main constituent of the inhibitory post-synaptic densities that anchors GABA_A_ receptors. The dynamic exchange between pools of extrasynaptic and synaptic Gephyrin is implicated GABA_A_R stabilization and synaptic strength (Petrini et al., 2014) and the clustering of Gephyrin can be affected during inhibitory synapse remodeling through CaMKII-dependent phosphorylation (Flores et al., 2015). Our functional assays using Gephyrin-GFP transfected hippocampal neurons indicated that short-term incubation with anti-Caspr2 LE IgGs induced a significant decrease in the density of synaptic Gephyrin clusters. However, the ratio of synaptic versus total Gephyrin-GFP clusters was not modified. We hypothesize that autoantibodies to Caspr2 may induce alterations of inhibitory synaptic contact and Gephyrin clustering at the post-synapse. Because of the dynamic exchange between the pools of synaptic and total Gephyrin, the clustering of total Gephyrin may be also perturbed. Thus our data suggest that Caspr2 autoantibodies from LE patients might induce structural alteration of the inhibitory post-synaptic scaffold by neutralizing Caspr2 function.

### Selectivity of Anti-Caspr2 Autoantibodies in the CSF and Serum of LE Patients

Autoantibodies have been reported to target selective modules of CAMs in peripheral neuropathies. For example, CIDP autoantibodies to Contactin are directed against functional modules implicated in its interaction with its glial partner Neurofascin155 and may thereby induce alteration of the paranodal complex (Ng et al., 2012; Labasque et al., 2014). Strikingly, in the present study, we showed that immunoreactivity of anti-Caspr2 CSF IgGs was restricted to the N-terminal discoïdin and LamininG1 domains in four out of seven LE patients suggesting that these two domains may play a major role in the physiopathology. The specific function of these N-terminal modules is still unknown, but they contain point mutations or deletions described in psychiatric, autism spectrum and language disorders associated with the Caspr2 gene, *cntnap2* (Zweier et al., 2009; O’Roak et al., 2011; Al-Murrani et al., 2012). We may hypothesize that these domains of Caspr2 could be implicated in its synaptic function. Indeed, we showed that the LE6 IgGs that selectively target the N-terminal modules display perturbing activity of the synaptic Gephyrin clusters. We may also notice that the anti-Caspr2 serum IgGs of LE patients bound the juxtaparanodes of mouse sciatic nerves whereas these patients did not present neuromyotonia. This may be either due to the restricted accessibility of juxtaparanodes or to the selectivity of the targeted N-terminal epitopes.

## Conclusion

This study highlights the role of inhibitory neurons as the main target for anti-Caspr2 autoantibodies and the potential role of the N-terminal discoïdin and LamininG1 domains. In patients with LE, anti-Caspr2 autoantibodies may alter Gephyrin clusters at inhibitory synaptic contacts possibly by disruption of Caspr2/TAG-1 interactions. All these data provide a clue to understand the central hyperexcitability observed in patients with autoimmune LE.

## Conflict of Interest Statement

The authors declare that the research was conducted in the absence of any commercial or financial relationships that could be construed as a potential conflict of interest.

## Supplementary Material

Figure S1 **Anti-Caspr2 IgGs of LE patients bind axons of hippocampal neurons in culture.** DIV7 hippocampal neurons were surface labeled with IgGs of LE2 **(A,B)**, LE3 **(C,D)**, LE4 **(E,F)**, LE5 **(G,H)** patients (red). Cells were fixed with 4% paraformaldehyde, permeabilized and double-stained for the somato-dendritic marker MAP2 (blue) or axonal tau (green). The LE IgGs did not bind hippocampal neurons when pre-adsorbed using incubation with Caspr2-transfected HEK cells **(B,D,F,H)**. Surface staining for Caspr2 using LE2–LE5 IgGs was restricted to tau-positive axons as indicated with yellow arrowheads in **(A,C,E,G)**. Single optical sections of confocal images. Bar is 7 μm.

Figure S2 **Anti-Caspr2 autoantibodies in LE patients label inhibitory axons in hippocampal cell culture.** Hippocampal neurons at DIV21 were surface labeled with IgGs of LE2 **(A)**, LE3 **(B)**, LE4 **(C)**, or LE5 **(D)** patients (red). Cells were fixed and permeabilized before double-staining for MAP2 (blue) and GAD65 (green). The overlay images and insets show that inhibitory axons and pre-synaptic terminals were immunostained with all patient’s autoantibodies. Single optical sections of confocal images. Bar is 10 μm; in insets, 3 μm.

## Abbreviations

CAM: cell adhesion molecule
CIDP: chronic inflammatory demyelinating polyneuropathy
DIV: day *in vitro*
DRG: dorsal root ganglia
EGF: epidermal growth factor-like
GPI: glycan phosphatidyl inositol
Ig: immunoglobulin
LE: limbic encephalitis
PDZ: PSD95/Dlg1/Zo-1
VGKC: voltage-gated potassium channels
