# Supplementary figures and images for: Inhibitory axons are targeted in hippocampal cell culture by anti-Caspr2 autoantibodies associated with limbic encephalitis
Source: Front Cell Neurosci. 2015 Jul 9;9:265. doi: 10.3389/fncel.2015.00265 (PMC4496579; doi:10.3389/fncel.2015.00265)

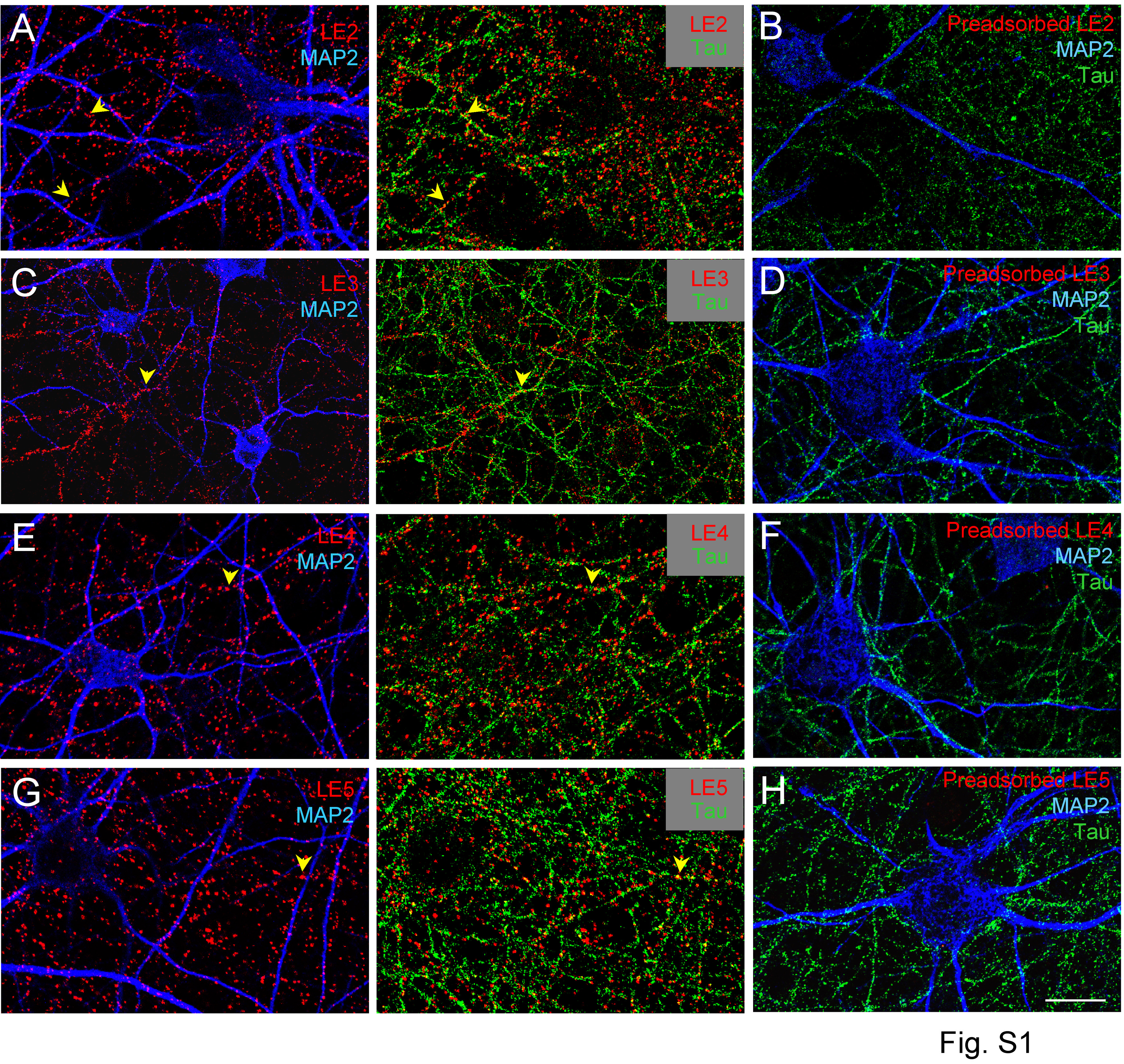

Supplement: Figure S1 — Anti-Caspr2 IgGs of LE patients bind axons of hippocampal neurons in culture. DIV7 hippocampal neurons were surface labeled with IgGs of LE2 (A,B), LE3 (C,D), LE4 (E,F), LE5 (G,H) patients (red). Cells were fixed with 4% paraformaldehyde, permeabilized and double-stained for the somato-dendritic marker MAP2 (blue) or axonal tau (green). The LE IgGs did not bind hippocampal neurons when pre-adsorbed using incubation with Caspr2-transfected HEK cells (B,D,F,H). Surface staining for Caspr2 using LE2–LE5 IgGs was restricted to tau-positive axons as indicated with yellow arrowheads in (A,C,E,G). Single optical sections of confocal images. Bar is 7 μm. [file Image_1.JPEG]

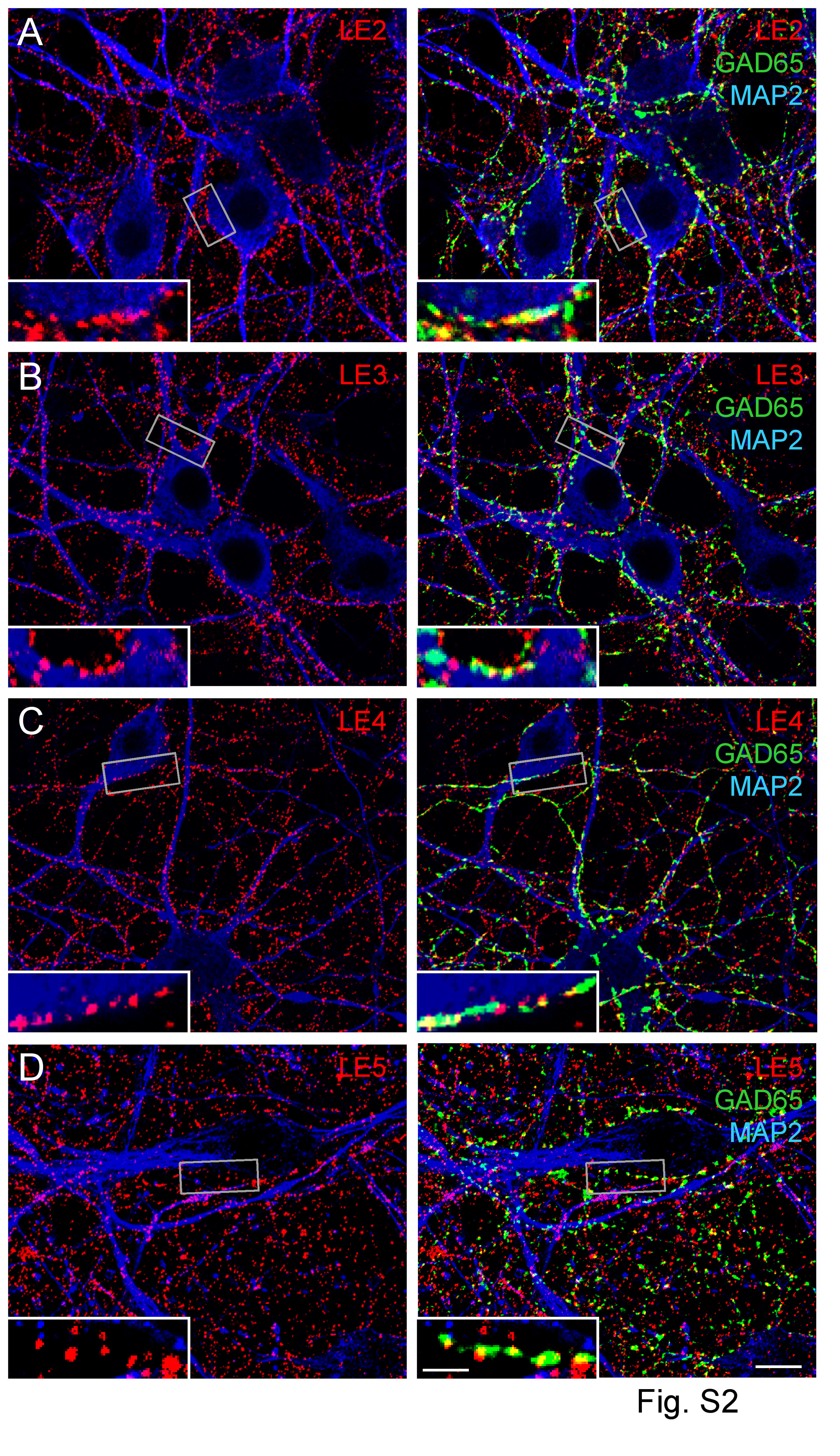

Supplement: Figure S2 — Anti-Caspr2 autoantibodies in LE patients label inhibitory axons in hippocampal cell culture. Hippocampal neurons at DIV21 were surface labeled with IgGs of LE2 (A), LE3 (B), LE4 (C), or LE5 (D) patients (red). Cells were fixed and permeabilized before double-staining for MAP2 (blue) and GAD65 (green). The overlay images and insets show that inhibitory axons and pre-synaptic terminals were immunostained with all patient’s autoantibodies. Single optical sections of confocal images. Bar is 10 μm; in insets, 3 μm. [file Image_2.JPEG]
